# Supplementary material for: Land use and semen quality: A fertility center cohort study
Source: PLoS One. 2021 Aug 12;16(8):e0255985. doi: 10.1371/journal.pone.0255985 (PMC8360504; doi:10.1371/journal.pone.0255985)
Supplement: S1 Fig — (DOCX) [file pone.0255985.s001.docx]

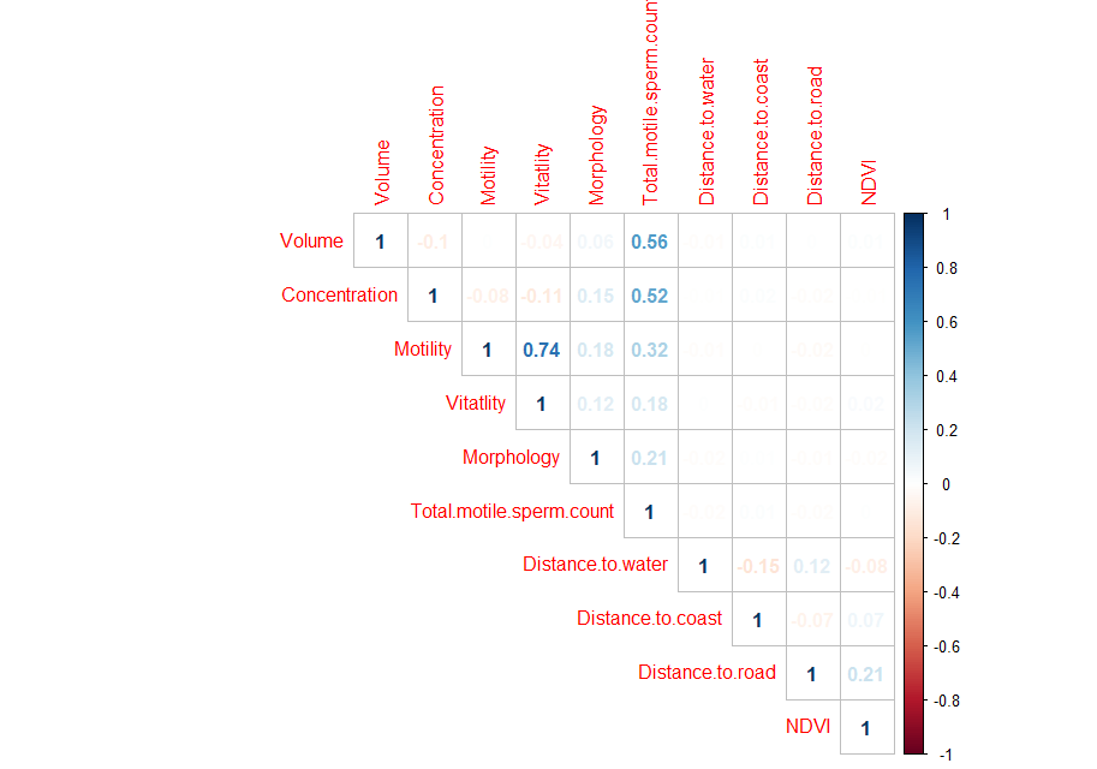


**S1 Fig. Pairwise correlation structure between four components of built environment and six sperm quality indicators.**
