# Supplementary figures and images for: Land use and semen quality: A fertility center cohort study
Source: PLoS One. 2021 Aug 12;16(8):e0255985. doi: 10.1371/journal.pone.0255985 (PMC8360504; doi:10.1371/journal.pone.0255985)

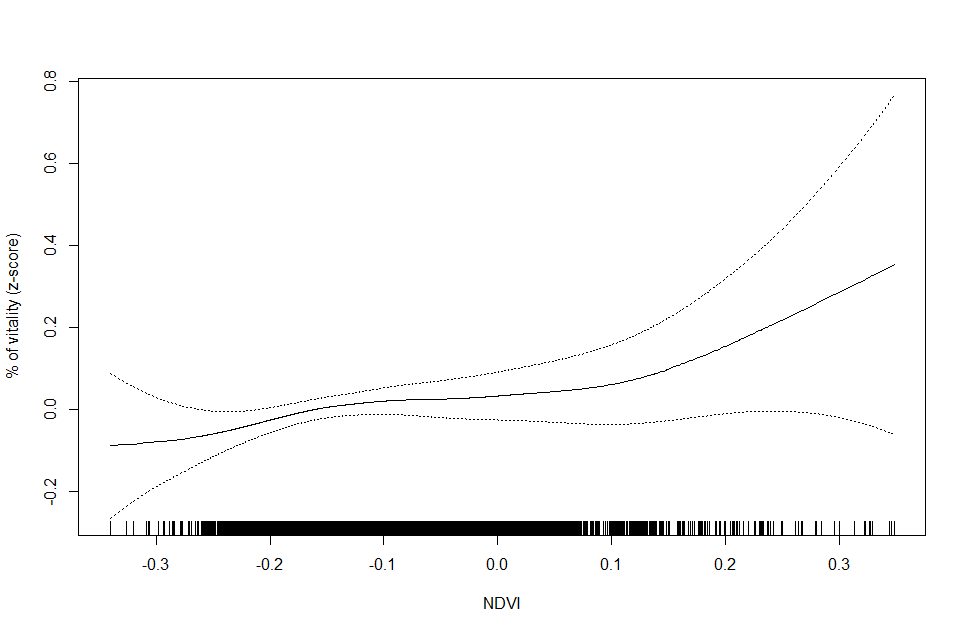


**S2 Fig. Association between NDVI within 500 m and % of sperm vitality in generalized additive model.**

Supplement: S2 Fig — (DOCX) [file pone.0255985.s002.docx]
