# Supplementary material for: Land use and semen quality: A fertility center cohort study
Source: PLoS One. 2021 Aug 12;16(8):e0255985. doi: 10.1371/journal.pone.0255985 (PMC8360504; doi:10.1371/journal.pone.0255985)
Supplement: S1 Table — Q1, lowest quartile; Q2, second quartile; Q3, third quartile; Q4, fourth quartile; NDVI, Normalized Difference Vegetation Index; Ph, P value for heterogeneity; Pt, P value for linear trend. Heterogeneity across quartiles was tested using Kruskal-Wallis rank sum test. Trend test was done with Kendall’s rank correlation test. Results with P value < 0.05 were bolded. (DOCX) [file pone.0255985.s003.docx]

**S1 Table. Mean value of semen quality indicators in each quartile of distance to fresh water, coast, and road with NDVI among 5,886 Korean infertile men.**

| Semen indicators | Distance to fresh water | | | | | |
| --- | --- | --- | --- | --- | --- | --- |
|  | Q1 (0―209.9m) | Q2 (209.9―382.8m) | Q3 (382.8―615.0m) | Q4 (615.0―1984.1m) | P^h^ | P^t^ |
| Volume (mL) | 3.1 ± 2.1 | 3.1 ± 1.8 | 3.0 ± 1.9 | 3.1 ± 1.6 | 0.828 | 0.698 |
| Count (million/mL) | 104.2 ± 71.2 | 102.9 ± 64.9 | 106.1 ± 72.1 | 103.9 ± 65.9 | 0.717 | 0.632 |
| Progressive motility (%) | **46.1 ± 12.9** | **44.8 ± 13.3** | **45.5 ± 13.4** | **45.9 ± 13.1** | **0.025** | 0.880 |
| Vitality (%) | 62.5 ± 12.7 | 62.3 ± 12.8 | 62.5 ± 12.4 | 63.0 ± 12.3 | 0.63 | 0.355 |
| Morphology (%) | 3.7 ± 1.8 | 3.7 ± 1.9 | 3.7 ± 1.8 | 3.7 ± 1.8 | 0.907 | 0.947 |
| Total motile sperm count (million) | 144.9 ± 120.8 | 139.7 ± 107.2 | 141.6 ± 106.4 | 143.9 ± 109.2 | 0.682 | 0.733 |
|  | Distance to coast | | | | | |
|  | Q1 (63.0-19837.4m) | Q2 (19837.4-24609.6m) | Q3 (24609.6-28785.6m) | Q4 (28785.6-79100.3m) | P^h^ | P^t^ |
| Volume (mL) | 3.1 ± 2 | 3 ± 2.4 | 3.1 ± 1.5 | 3.1 ± 1.5 | 0.099 | 0.280 |
| Count (million/mL) | 102.3 ± 65.9 | 104.1 ± 65.9 | 105.3 ± 67.3 | 105.7 ± 71.8 | 0.664 | 0.301 |
| Progressive motility (%) | 45.8 ± 13.4 | 45.2 ± 12.8 | 46.0 ± 12.7 | 45.5 ± 13.4 | 0.344 | 0.937 |
| Vitality (%) | 62.9 ± 12.5 | 62.6 ± 12.5 | 62.7 ± 12 | 62.2 ± 12.9 | 0.746 | 0.272 |
| Morphology (%) | 3.6 ± 1.8 | 3.7 ± 1.8 | 3.7 ± 1.8 | 3.7 ± 1.8 | 0.637 | 0.383 |
| Total motile sperm count (million) | 143.3 ± 111.2 | 141.3 ± 123.2 | 145.2 ± 109 | 146.4 ± 110.2 | 0.268 | 0.214 |
|  | Distance to major roadway | | | | | |
|  | Q1 (5.1―184.8m) | Q2 (184.8―495.7m) | Q3 (495.7―1295.9m) | Q4 (1295.9―58607.8m) | P^h^ | P^t^ |
| Volume (mL) | 3.1 ± 2.1 | 3.1 ± 1.9 | 3.1 ± 1.5 | 3.1 ± 1.8 | 0.907 | 0.537 |
| Count (million/mL) | 104.9 ± 68.2 | 104.7 ± 67.1 | 103.7 ± 68.9 | 103.8 ± 70.1 | 0.677 | 0.334 |
| Progressive motility (%) | **45.4 ± 13.5** | **46.2 ± 12.7** | **45.8 ± 13.2** | **44.9 ± 13.3** | **0.033** | 0.510 |
| Vitality (%) | 62.2 ± 12.8 | 63.1 ± 12.4 | 62.6 ± 12.4 | 62.5 ± 12.5 | 0.263 | 0.298 |
| Morphology (%) | 3.7 ± 1.8 | 3.7 ± 1.9 | 3.7 ± 1.8 | 3.6 ± 1.9 | 0.374 | 0.767 |
| Total motile sperm count (million) | 142.9 ± 119.8 | 144.9 ± 109.3 | 143.0 ± 109.7 | 139.3 ± 104.7 | 0.420 | 0.602 |
|  | NDVI within 500m | | | | | |
|  | Q1 (-0.34― -0.20) | Q2 (-0.20― -0.15) | Q3 (-0.15― -0.08) | Q4 (-0.08―0.35) | P^h^ | P^t^ |
| Volume (mL) | 3.06 ± 1.88 | 3.11 ± 2.28 | 3.07 ± 1.51 | 3.05 ± 1.54 | 0.955 | 0.974 |
| Count (million/mL) | 104.26 ± 66.35 | 106.35 ± 69.78 | 104.15 ± 70.1 | 102.28 ± 68.04 | 0.233 | 0.113 |
| Progressive motility (%) | 45.35 ± 13.04 | 45.91 ± 13.08 | 45.4 ± 13.41 | 45.61 ± 13.21 | 0.458 | 0.722 |
| Vitality (%) | 62.13 ± 12.42 | 62.81 ± 12.43 | 62.54 ± 12.77 | 62.81 ± 12.5 | 0.165 | 0.118 |
| Morphology (%) | 3.74 ± 1.82 | 3.64 ± 1.8 | 3.73 ± 1.87 | 3.6 ± 1.83 | 0.070 | 0.094 |
| Total motile sperm count (million) | 138.86 ± 102.73 | 148.12 ± 120.87 | 143.07 ± 113.01 | 139.93 ± 106.43 | 0.153 | 0.595 |

Q1, lowest quartile; Q2, second quartile; Q3, third quartile; Q4, fourth quartile; NDVI, Normalized Difference Vegetation Index; P^h^, P value for heterogeneity; P^t^, P value for linear trend. Heterogeneity across quartiles was tested using Kruskal-Wallis rank sum test. Trend test was done with Kendall's rank correlation test. Results with P value < 0.05 were bolded.
