# Supplementary material for: Land use and semen quality: A fertility center cohort study
Source: PLoS One. 2021 Aug 12;16(8):e0255985. doi: 10.1371/journal.pone.0255985 (PMC8360504; doi:10.1371/journal.pone.0255985)
Supplement: Attachment — Submitted filename: Response to reviewer R1_R2 (2021-07-07).docx [file pone.0255985.s004.docx]

PONE-D-21-13270

Land use and semen quality: a fertility center cohort study

PLOS ONE

Dear Dr. Stefan Schlatt,

Thank you for your and reviewers’ comments. We revised the manuscript following the suggestions. We will look forward to your feedback.

Thank you,

Dae Keun Kim

**Responses to editorial review**

PONE-D-21-13270R1

Land use and semen quality: a fertility center cohort study

Response to editorial review:

**Comment #1**: Please upload a Response to Reviewers letter which should include a point by point response to each of the points made by the Editor and / or Reviewers. (This should be uploaded as a 'Response to Reviewers' file type.) Please follow this link for more information: http://blogs.PLOS.org/everyone/2011/05/10/how-to-submit-your-revised-manuscript/

**Response #1**: We are grateful for the editorial comments. The manuscript was revised accepting all the suggestions.

**Comment #2**: We note that Supplemental Figure 1 in your submission contain map images which may be copyrighted. All PLOS content is published under the Creative Commons Attribution License (CC BY 4.0), which means that the manuscript, images, and Supporting Information files will be freely available online, and any third party is permitted to access, download, copy, distribute, and use these materials in any way, even commercially, with proper attribution. For these reasons, we cannot publish previously copyrighted maps or satellite images created using proprietary data, such as Google software (Google Maps, Street View, and Earth). For more information, see our copyright guidelines: http://journals.plos.org/plosone/s/licenses-and-copyright.

We require you to either (1) present written permission from the copyright holder to publish these figures specifically under the CC BY 4.0 license, or (2) remove the figures from your submission:

3.1. You may seek permission from the original copyright holder of Supplemental Figure 1 to publish the content specifically under the CC BY 4.0 license.

We recommend that you contact the original copyright holder with the Content Permission Form (http://journals.plos.org/plosone/s/file?id=7c09/content-permission-form.pdf) and the following text:

“I request permission for the open-access journal PLOS ONE to publish XXX under the Creative Commons Attribution License (CCAL) CC BY 4.0 (http://creativecommons.org/licenses/by/4.0/). Please be aware that this license allows unrestricted use and distribution, even commercially, by third parties. Please reply and provide explicit written permission to publish XXX under a CC BY license and complete the attached form.”

Please upload the completed Content Permission Form or other proof of granted permissions as an "Other" file with your submission.

In the figure caption of the copyrighted figure, please include the following text: “Reprinted from [ref] under a CC BY license, with permission from [name of publisher], original copyright [original copyright year].”

3.2. If you are unable to obtain permission from the original copyright holder to publish these figures under the CC BY 4.0 license or if the copyright holder’s requirements are incompatible with the CC BY 4.0 license, please either i) remove the figure or ii) supply a replacement figure that complies with the CC BY 4.0 license. Please check copyright information on all replacement figures and update the figure caption with source information. If applicable, please specify in the figure caption text when a figure is similar but not identical to the original image and is therefore for illustrative purposes only.

The following resources for replacing copyrighted map figures may be helpful:

USGS National Map Viewer (public domain): http://viewer.nationalmap.gov/viewer/

The Gateway to Astronaut Photography of Earth (public domain): http://eol.jsc.nasa.gov/sseop/clickmap/

Maps at the CIA (public domain): https://www.cia.gov/library/publications/the-world-factbook/index.html and https://www.cia.gov/library/publications/cia-maps-publications/index.html

NASA Earth Observatory (public domain): http://earthobservatory.nasa.gov/

Landsat: http://landsat.visibleearth.nasa.gov/

USGS EROS (Earth Resources Observatory and Science (EROS) Center) (public domain): http://eros.usgs.gov/#

Natural Earth (public domain): http://www.naturalearthdata.com/

**Response #2**: As addressed in our prior response, the figure was created using ArcGIS. However, to avoid potential issues from the readers about the copyrights, we removed the supplemental figure 1 as suggested.

**Comment #3**: Please amend your list of authors on the manuscript to ensure that each author is linked to an affiliation.

We note that you have included affiliation numbers 1 - 8 however only affiliations 1-7 have authors linked to them. Please amend affiliation 8 to link an author to it or remove if added in error.

**Response #3:** Thanks to the comments, we corrected the affiliation statement (p.1 in the revised manuscript).

**Comment #4**: Thank you for including your ethics statement on the online submission form: "The study design was approved by the institutional review board of Gangnam CHA hospital (GCI-18-48). As a research involving the retrospective review, this study qualified a waiver of informed consent.". To help ensure that the wording of your manuscript is suitable for publication, would you please also add this statement at the beginning of the Methods section of your manuscript file.

**Response #4**: We added the statement in the revised manuscript (line 71-73, p. 4).

Reviewers' comments:

**Reviewer #1**

 The authors report on 5886 men presenting for infertility evaluation in whom various markers of the ‘built-up environment’ were assessed for their impact on semen quality. Specifically, the distance to fresh-water, to the coast and to major roadways, and the neighbourhood ‘greenness index’, described as the Normalized Difference Vegetation Index [NDVI] as assessed from satellite data. A single semen analysis used during infertility evaluation was assessed for various routine parameters.

The great majority of the patient base were white collar workers with an average age of 39 and a normal- overweight BMI. Smoking rates are very high in this population of 51%. These matters were considered in the analyses.

The principle conclusion was that there was no clear association between parameters of the built-up environment and semen quality other than for the NDVI and sperm vitality. There was also some relationship between proximity to fresh water and semen quality, and of sperm vitality and distance from major roadways. All these data point to some association between the relative ‘greenness of the environment’ and semen quailty amongst men with infertility.

The authors rightly acknowledge the limitations of the study and the mechanism by which any effects may occur remains obscure and a matter of speculation. This study is intriguing and in broad terms points toward the need to understand the environment and lifestyle impact on semen quality and to take measures to ensure that the environment and lifestyle factors are optimised to promote fertility. In the Korean population, the attention clearly needs to be paid to the extraordinary smoking rates which would be deleterious to semen quality.

I have a few minor comments:

**Comment #1**: In terms of proximity to water, the question rises to what type of water? They mention rivers and lakes. Can these vary in the quality of the water in those sites, for example, are there any which are heavily polluted and therefore, amongst men proximal to those, might beneficial effects pf water proximity disappear?

**Response #1**: We are thankful for the reviewer’s comments. There might have been hazardous effect by water pollution in some areas. According to the study of Mainali et al. (2018), with temporal and spatial variation in different months of each season, water quality of the river basin of Korea exceeded ‘poor’ category up to 15 percent of times between 2012 and 2016. Given the relatively small proportion of water pollution in the area, we believe that the potential misclassification (benefit of proximity to fresh water) would have minimally biased the result. We discussed this issue (line 248-253, p.16)

**Comment #2**: The data description I think distances can be expressed to the nearest metre, for example, 486.7m can fairly be rounded to 487m for the purposes of this discussion to make the figures easier to read.

**Response #2**: We revised the Table 1 using rounded numbers for the distances (Table 1).

**Comment #3**: The effects do not appear to be dose related. Similarly, when they asked about smoking, it was a Yes or No question. Is it possible to consider those who are very heavy smokers as opposed to minimal smokers and see if there might be some interaction which was not taken into account in the existing statistical approach?

**Response #3**: There might have been an interaction by amount of smoking in the association between built environment and semen quality. As there is no information about the amount of smoking, we mentioned this issue as one of the limitation (line 256, p.17).

**Comment #4**: Line 191 – They talk about motility being highest in the second quartile, distance from roadway and lowest in the fourth quartile. Why could such a relationship be so non-linear, in other words, why is the first quartile lower than the second? Theoretical reasons might underlie that observation.

**Response #4**: The non-linear association of proximity to fresh water or roadway with semen quality in our study may be explained by U-shaped association between built environment and active traveling which is reported in Liu (2021). We added this discussion (line 134-136, p.16)
